# The association between visual impairment and mental disorders

**DOI:** 10.1038/s41598-024-52389-6

**Published:** 2024-01-27

**Authors:** Alireza Hashemi, Hassan Hashemi, Alireza Jamali, Hamed Ghasemi, Fatemeh Ghazizadeh Hashemi, Mehdi Khabazkhoob

**Affiliations:** 1 Noor Research Center for Ophthalmic Epidemiology, Noor Eye Hospital, Tehran, Iran; 2 Noor Ophthalmology Research Center, Noor Eye Hospital, Tehran, Iran; 3 Rehabilitation Research Center, Department of Optometry, School of Rehabilitation Sciences, Iran University of Medical Sciences, Tehran, Iran; 4 Eye Research CenterFarabi Eye Hospital, Tehran University of Medical Sciences, Tehran, Iran; 5 Psychiatry and Psychology Research CenterRoozbeh Hospital, Tehran University of Medical Sciences, Tehran, Iran; 6 Department of Basic Sciences, School of Nursing and Midwifery, Shahid Beheshti University of Medical Sciences, Tehran, Iran

**Keywords:** Medical research, Neurology

## Abstract

To determine the association between visual impairment (VI) and some mental disorders using the general health questionnaire (GHQ) in individuals aged 60 years and above in Tehran, Iran. The present population-based cross-sectional study was conducted on people aged 60 and older in Tehran, Iran using multi-stage cluster sampling. After selecting the samples, examinations including visual acuity measurement, refraction, and slit-lamp biomicroscopy were performed for all participants. The GHQ was used to examine mental disorders. Simple and multiple linear regressions were used to investigate the association between VI and mental disorders. Of the 3740 invitees, 3310 participated in the study (response rate: 88.50%). After applying the exclusion criteria, 2789 individuals were analyzed for this report. Mean score of physical, anxiety, social and depression disorders in people with and without VI was 3.74 ± 2.03, 5.81 ± 2.79, 7.56 ± 1.91, 1.32 ± 1.90, and 3.14 ± 1.76, 4.93 ± 2.71, 8.09 ± 1.99, 0.91 ± 1.38, respectively. The total score of GHQ in participants with and without VI was 18.43 ± 4.75 and 17.07 ± 4.19, respectively. The association between GHQ subscales and total GHQ score with VI by multiple linear regression showed that VI had a statistically significant direct association with physical symptoms (β = 0.37; 95% CI 0.12 to 0.62) and anxiety (β = 0.48; 95% CI 0.16 to 0.81). Nevertheless, depression had a borderline association with VI (β = 0.21; 95% CI − 0.03 to 0.45) and social dysfunction did not have a statistically significant association with VI in the final linear regression model and did not remain in the model. Total GHQ score had a statistically significant association with VI (β = 1.02; 95% CI 0.39 to 1.64) in the presence of covariates. Regarding the association between VI and GHQ components, the physical symptoms had the highest R^2^ (R^2^ = 0.159). Patients with VI suffer more from anxiety, depression, and physical symptoms regardless of age, sex, education, and other effective variables. The coincidence of aging with VI and the association of VI with mental disorders emphasizes the importance of a broader view of the elderly and the aging process.

## Introduction

Visual impairment (VI) is one of the most common preventable disorders. Globally, 1.1 billion people were living with vision loss in 2020; of which 43 million were blind, 295 million had moderate to severe VI, 258 million had mild VI, and 510 million had near vision problems^1^. However, these figures would be expected to increase significantly in the future due to population aging and lifestyle changes. The projections indicate that there will be 61 million blind people, 474 million people with moderate to severe VI, and 360 million people with mild VI in 2050, this is while 90% of the cases with VI live in low- and middle-income countries^1^.

In addition to the significant economic burden estimated at more than $3 trillion globally directly and indirectly, VI affects various aspects of a person’s life. Studies showed that individuals with VI are more prone to falls^2,3^. Moreover, these people have reduced ability to perform daily tasks^4^, reduced quality of life^5^, lower self-rated health^6^, reduced physical activity^7^, and impaired mental health^8^. VI is one of the major risk factors for functional status decline in community-living people^9^. Since the elderly usually have several co-existing problems, their health is more at risk leading to a further decrease in quality of life, disability, increased healthcare costs, and increased hospitalization and mortality^10–13^.

So far, some studies have investigated the association between VI and mental disorders such as depression^14^, anxiety^15^, poor perceived health^6^, suicide^16^, and cognitive impairment^17^. VI is usually conceptualized as a physical problem; so, its psychological consequences have received less attention^18^. Studies investigating the association between VI and psychological problems were mostly related to Western^8,11,16^ and high-income countries^3,4,10^; On the other hand, most of the studies conducted in Eastern countries were hospital-based^17^ or limited to a group of patients with specific ocular diseases.

The increase in life expectancy and the world population aging has led health policymakers to pay special attention to various aspects of health, especially vision in the elderly population. Accordingly, various studies on the elderly population have been designed and implemented in Iran^19–22^; however, limited hospital-based studies evaluated the psychological consequences of VI^23^. To our best knowledge, there is no population-based study in this regard in Iran. Considering the lack of evidence and the need for information for health planning, the present report aimed to evaluate the association between VI and mental disorders using the general health questionnaire (GHQ).

## Methods

Tehran geriatric eye study (TGES) is a population-based cross-sectional study conducted in 2019 in the metropolis of Tehran (the capital of Iran) on 3310 people aged 60 years and above using multi-stage stratified random cluster sampling. The current project was carried out with the aim of investigating VI in the elderly of Tehran. For this reason, the sample size was calculated based on the prevalence of VI in the elderly. Considering a prevalence of 5.2% for VI as the main objective of the study, precision of 1%, and confidence interval of 95%, the sample size was estimated at 1894 subjects. After applying a design effect of 1.5 and a non-response rate of 10% (sample size/(1-non-response rate)), the sample size was calculated at 3155 subjects, which was rounded up to 3200 participants.

In the first stage, each of the 22 municipality districts of Tehran was considered a stratum, and the population aged 60 years and above in each district was obtained from the National Statistics Center. The population to be selected from each district was determined proportionally to the size. Next, a block map of each district was prepared, and each block was considered a cluster. Finally, a total of 160 blocks were randomly selected from all 22 districts of Tehran. The number of clusters in each district was proportional to its population and each cluster contained 20 individuals. After the selected blocks were identified, a sampling team went to their addresses and the first house was chosen as the cluster head by being located on the southwest side of the selected block. The next households were selected in a counter-clockwise movement, and all people aged 60 years and older were invited to participate in the study after explaining the objectives of the study and ensuring the confidentiality of the data. If someone was willing to participate in the study, informed consent was obtained and an ID card was issued. Study participants were transferred to the examination site (Noor Eye Hospital, Tehran, Iran) free of charge.

Once the study subjects were presented to the examination site, complete demographic, socio-economic, psychometric, and anthropometric information was collected by a trained person through standard questionnaires, followed by laboratory tests, and optometric and ophthalmologic examinations. All optometric examinations were performed by two experienced optometrists in a room with standard illumination. The two examiners showed a high agreement in measuring uncorrected visual acuity (ICC: 0.994) and the spherical equivalent (SE) of subjective refraction (ICC: 0.967) in a pilot of 30 individuals.

Optometric examinations included measurement of uncorrected distance visual acuity (UCVA) using an LED chart (Smart LC 13, Medizs Inc., Korea) at 6 m, objective refraction using an autorefractometer-keratometer (ARK-510A, Nidek Co. LTD, Aichi, Japan), and subjective refraction to determine optimal distance optical correction and the best-corrected distance visual acuity (BCVA). Then, a complete anterior and posterior segment ocular examination was performed by an ophthalmologist using a slit-lamp biomicroscope (B900, Haag-Streit AG, Bern, Switzerland) and a + 90 diopter (D) lens.

### General health questionnaire (GHQ)

The GHQ was designed by Goldberg and Hillier aiming at investigating the psychological aspects of quality of life. This questionnaire consists of 28 questions and four subscales including physical symptoms (questions 1–7), anxiety and insomnia (questions 8–14), social dysfunction (questions 15–21), and depression (questions 22–28). Each question is scored on a 4-point (0–3) Likert scale. The scores for the subscales are 0–21 and the scores of the total scale are 0–84. The validity and reliability of GHQ have already been confirmed in the Iranian population^24^.

According to previous studies^25^, Cronbach’s alpha coefficients of the GHQ total score was 0.90, and this coefficient for Depression, Anxiety and insomnia, Social dysfunction and Physical symptoms were 0.88, 0.81, 0.78 and 0.69, respectively.

The Mini-Mental State Examination (MMSE) was also used to investigate dementia; individuals with severe dementia were excluded from this report as their subjective findings may not be valid. The interviews were conducted by an expert psychologist to complete the GHQ and MMSE questionnaires.

### Study variables

Diabetes was defined based on HbA1c > %6.4 or blood sugar (BS) > 200 mg/dl. To determine the economic status, the data of 13 household assets were collected, and a wealth index was produced using principal component analysis according to the weight of the first component; then an economic status variable with three categories (low, moderate, high) was created. Also, the history of smoking and alcohol consumption was collected by self-report.

VI was defined based on the presenting visual acuity (PVA) according to the WHO guideline and categorized into low vision and blindness. For the participants not wearing corrective spectacles, PVA is equivalent to UCVA; for those wearing spectacles, PVA is equivalent to visual acuity with current spectacles. The low vision was defined as a PVA between 0.5 LogMAR (20/60) and ≤ 1.3 LogMAR (20/400) in the better-seeing eye. Blindness was defined as a PVA worse than 1.3 LogMAR (20/400) in the better-seeing eye. In this study, considering that completing the GHQ questionnaire requires cognitive health, people with cognitive problems were excluded from the study.

### Statistical analysis

This analysis was conducted using Stata v 12.0 (Stata Corporation, TX, USA). To estimate the age and sex-standardized prevalence, the population of Tehran was prepared and then the sample was weighted based on it. All point estimates were directly standardized for age and sex in population of Tehran. The design effect of a cluster sampling approach was taken into consideration and adjusted for in the calculation of 95% CIs (linearized standard error). The 95% CIs were calculated by assuming normal approximation.

Next, the age-sex standardized prevalence of visual impairment and 95% confidence interval (CI) were reported. The overall GHQ score and GHQ subscale scores were compared between two groups with and without VI using independent samples t-test and chi-square test. Levene's test was used to check the homogeneity of variances and its results were reported. Simple and multiple linear regression was used to investigate the association between VI and GHQ and its subscales.

For these models, the covariates of age, sex, education, body mass index, diabetes, alcohol consumption, and economic status were entered into the model and then entered into the final model using the Backward method. The final model was considered a model where all variables are significant. In the final model, lack of multicollinearity was also checked and if there was collinearity between the variables with variance inflation factor (VIF) greater than 2, one of them was removed from the model. Also, other assumptions of linear regression such as lack of outlier, linearity, normality were also investigated. In this study, P-value < 0.05 was considered statistically significant.

### Ethical issues

The study protocol was approved by the Ethics Committee of the National Institute for Medical Research Development (NIMAD) under the supervision of the Iran Ministry of Health and Medical Education. Tenets of the Helsinki Declaration were observed in this study and written informed consent was obtained from all participants (ethical code: IR.NIMAD.REC.1397.292).

### Ethics approval and consent to participate

Informed consent was obtained from all participants. The principles of the Helsinki Declaration were followed in all stages of this study. The protocol of the study was approved by the Ethics Committee of the National Institute for Medical Research Development (NIMAD) under the auspices of the Iranian Ministry of Health.

## Results

**Figure 1:**
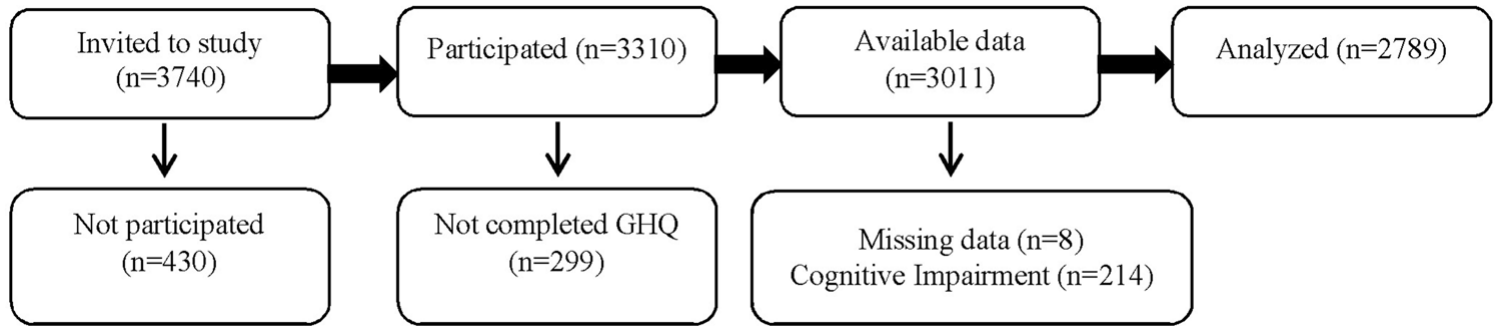
Follow chart of present study.

Of the 3740 invitees, 3310 participated in TGES (response rate: 88.5%). Of these, 3011 completed the GHQ questionnaire. Eight individuals were excluded from the analysis due to missing cognitive data and 214 individuals were excluded due to cognitive problems. Finally, statistical analysis was performed on the data of 2789 participants (Fig. 1).

The mean age of the participants analyzed was 68.91 ± 7.12 years (range: 60 to 97) and 1680 (52.5%) were female. 352 individuals (12.9%) had university education and 919 (34.42%) had high economic status. Table 1 presents the distribution of demographic variables and GHQ subscales in the present study. Age and sex-standardized prevalence of VI, low vision, and blindness was 12.59% (95% CI 11.35 to 13.82), 11.83% (95% CI 10.63 to 13.03), and 0.75% (95% CI 0.43 to 1.07), respectively.

**Table 1:** Mean and 95% confidence interval for general health questionnaire (GHQ) scales and demographic variables.

	Variables		n	Estimate	95% CI*
Percent	Education	Illiterate	339	13.1	13.09 (10.51 to 16.19)
		Primary school	869	30.9	30.92 (27.59 to 34.46)
		Guidance school	532	18.6	18.58 (16.65 to 20.69)
		High school	697	24.5	24.50 (21.76 to 27.45)
		College	352	12.9	12.90 (10.56 to 15.69)
	Gender	Male	1109	47.5	47.46 (44.27 to 50.68)
		Female	1680	52.5	52.54 (49.32 to 55.73)
	diabetes	No	1971	71.9	71.90 (69.93 to 73.79)
		Yes	805	28.1	28.10 (26.21 to 30.07)
	Smoking	No	2431	85.3	85.31 (83.88 to 86.63)
		Yes	358	14.7	14.69 (13.37 to 16.12)
	Alcohol	No	2637	94.89	93.96 (92.47 to 95.17)
		Yes	152	5.45	6.04 (4.83 to 7.53)
	Visual impairment	No	2438	87.41	86.05 (84.18 to 87.73)
		Yes	351	12.59	13.95 (12.27 to 15.82)
	Low vision	No	2459	88.17	86.82 (85.00 to 88.45)
		Yes	330	11.83	13.18 (11.55 to 15.00)
	Blindness	No	2768	99.25	99.23 (98.81 to 99.50)
		Yes	21	0.75	0.77 (0.50 to 1.19)
	Economic status	Low	960	32.95	35.56 (32.13 to 39.13)
		Moderate	910	32.62	32.29 (30.23 to 34.42)
		High	919	34.42	32.15 (28.45 to 36.10)
Mean	Age (yrs. old)		2789	68.01	68.91 (68.51 to 69.32)
	BMI (Kg/m^2^)		2776	28.76	28.43 (28.22 to 28.63)
	GHQ scales				
	Physical (score)		2789	3.32	3.22 (3.13 to 3.32)
	Anxiety (score)		2789	5.15	5.05 (4.91 to 5.20)
	Social (score)		2789	8.06	8.01 (7.85 to 8.18)
	Depression (score)		2789	0.97	0.97 (0.88 to 1.05)
	Total (score)		2789	17.5	17.26 (17.03 to 17.49)

*The design effect of a cluster sampling approach was taken into consideration and adjusted for in the calculation of 95% CIs (linearized standard error).

Table 2 shows the mean score of GHQ subscales in participants with and without VI. As seen in Table 2, the mean scores of physical symptoms, anxiety, social dysfunction, and depression in participants without VI were 3.14 ± 1.76, 4.93 ± 2.71, 8.09 ± 1.99, and 0.91 ± 1.38, and in those with VI were 3.74 + 2.03, 5.81 + 2.79, 7.56 + 1.91, 1.32 + 1.90, respectively. According to the independent samples *t* test, the mean total GHQ score (P < 0.001) and the mean scores of physical symptoms (P < 0.001), anxiety and insomnia (P < 0.001), and depression (P < 0.001) were significantly higher in participants with VI while the social dysfunction score was significantly higher in those without VI (P < 0.001). The status of other variables, including background and predisposing variables, is presented in Table 2.

**Table 2:** Comparison of general health questionnaire (GHQ) scales, background and predisposing variables between in patients with visual impairment.

Variables			Visual impairment		p-value**	p-value***
			No	Yes		
			Mean ± SD*	Mean ± SD		
GHQ scales	Anxiety (score)		4.93 ± 2.71	5.81 ± 2.79	< 0.001	< 0.001
	Social (score)		8.09 ± 1.99	7.56 ± 1.91	< 0.001	< 0.001
	Depression (score)		0.91 ± 1.38	1.32 ± 1.9	< 0.001	< 0.001
	Total (score)		17.07 ± 4.19	18.43 ± 4.75	< 0.001	< 0.001
Background variables	Age (yrs. old)		68.28 ± 6.67	72.81 ± 8.48	< 0.001	< 0.001
	Sex (male)		968 (39.7%)	141 (40.2%)	0.861	
	Education	Illiterate	258 (10.6%)	81 (23.1%)	< 0.001	
		Primary school	738 (30.3%)	131 (37.3%)		
		Guidance school	467 (19.2%)	65 (18.5%)		
		High school	643 (26.4%)	54 (15.4%)		
		College	332 (13.6%)	20 (5.7%)		
Predisposing variables	BMI (Kg/m^2^)		28.54 ± 4.63	27.74 ± 4.75	< 0.001	< 0.001
	Smoking [yes]		312 (12.8%)	46 (13.1%)	0.872	
	Diabetes [yes]		690 (28.4%)	115 (33.2%)	0.063	
	Alcohol [yes]		138 (5.7%)	14 (4.0%)	0.197	
	Economic status	Low	781 (32.0%)	179 (51.0%)	< 0.001	
		Moderate	798 (32.7%)	112 (31.9%)		
		High	859 (35.2%)	60 (17.1%)		

*SD* standard deviation.

*The design effect of a cluster sampling approach was taken into consideration and adjusted for in the calculation of standard deviation.

**P-value was calculated by independent t-test.

***P-value was calculated by Levene’s Test for equality of variances.

The mean scores of physical symptoms, anxiety and insomnia, social dysfunction, and depression in participants without blindness were 3.31 ± 1.83, 5.15 ± 2.74, 8.07 ± 1.98, and 0.97 ± 1.48, and in those with blindness were 3.52 ± 2.11, 5.62 ± 1.86, 7.67 ± 2.35, and 0.86 ± 1.15, respectively. There was no statistically significant difference in the mean total GHQ score and GHQ subscales between the two groups (all P values > 0.05).

The association between GHQ subscales and total GHQ score with VI was investigated by simple and multiple linear regression. The final results of each model based on the multiple model after controlling the effect of covariates are shown in Table 3. According to Table 3, VI had a statistically significant direct association with physical symptoms (β = 0.37; P = 0.004) and anxiety (β = 0.48; P = 0.004). However, depression had a borderline association with VI (β = 0.21; P = 0.083), as can be seen in Table 3, social dysfunction does not have a statistically significant association with VI and did not remain in the model. The results of Table 3 show that in the presence of other covariates, total GHQ score has a statistically significant association with VI. Table 3 shows the standardized regression coefficients and R2. As can be seen, regarding the association between VI and GHQ components, the physical symptoms component has the highest R^2^.

**Table 3:** The association between visual impairment with general health questionnaire (GHQ) and their scales in multiple linear regressions.

		Physical	Anxiety	Social	Depression	Total GHQ
		B (95% CI*);Bs; p-value	B (95% CI); Bs; p-value	B (95% CI); Bs; p-value	B (95% CI); Bs; p-value	B (95% CI); Bs; p-value
Visual impairment		0.37 (0.12 to 0.62); 0.05; 0.004	0.48 (0.16 to 0.81); 0.06; 0.004	NR	0.21 (− 0.03 to 0.45); 0.04; 0.083	1.02 (0.39 to 1.64); 0.07; 0.002
Age		NR	NR	NR	NR	− 0.03 (− 0.06 to 0.00); − 0.03; 0.051
Female sex		1.26 (1.10 to 1.41); 0.34; < 0.001	1.31 (1.09 to 1.52); 0.23; < 0.001	0.21 (0.01 to 0.41); 0.07; 0.041	0.17 (0.04 to 0.3); 0.06; 0.012	2.9 (2.51 to 3.30); 0.34; < 0.001
Diabetes		0.24 (0.08 to 0.39); 0.05; 0.003	0.43 (0.19 to 0.67); 0.07; < 0.001	NR	NR	0.60 (0.21 to 0.99); 0.07; 0.003
Smoking		0.22 (0.01 to 0.43); 0.04; 0.041	0.57 (0.25 to 0.9);0.07; < 0.001	NR	0.29 (0.12 to 0.46); 0.07; < 0.001	1.1 (0.61 to 1.6); 0.08; < 0.001
Alcohol		− 0.27 (− 0.53 to − 0.01); − 0.03; 0.042	NR	0.48 (0.11 to 0.84); 0.06; 0.011	NR	NR
BMI		NR	NR	NR	NR	NR
Socio economic	Low	Reference	Reference	Reference	Reference	Reference
	Middle	− 0.07 (− 0.24 to 0.1); − 0.01; 0.445	− 0.32 (− 0.61 to − 0.03); − 0.04; 0.030	0.2 (0 to 0.41); 0.04; 0.052	− 0.17 (− 0.33 to − 0.01); − 0.05; 0.036	− 0.43 (− 0.9 to 0.05); − 0.04; 0.076
	High	− 0.28 (− 0.46 to − 0.1); − 0.07; 0.002	− 0.74 (− 1.05 to − 0.42); − 0.13; < 0.001	0.56 (0.32 to 0.8); 0.14; < 0.001	− 0.34 (− 0.51 to − 0.16); − 0.12; < 0.001	− 0.88 (− 1.35 to − 0.40); − 0.09; < 0.001
Education	Illiterate	Reference	Reference	Reference	Reference	Reference
	Primary school	− 0.25 (− 0.53 to 0.02); − 0.07; 0.069	− 0.15 (− 0.54 to 0.24); − 0.3; 0.446	0.12 (− 0.19 to 0.44); 0.04; 0.445	− 0.19 (− 0.47 to 0.08); − 0.06; 0.168	− 0.53 (− 1.19 to 0.13); − 0.05; 0.116
	Guidance School	− 0.58 (− 0.87 to − 0.3); − 0.13; < 0.001	− 0.51 (− 0.94 to − 0.08); − 0.07; 0.020	0.68 (0.33 to 1.04); 0.13; < 0.001	− 0.42 (− 0.68 to − 0.16); − 0.11; 0.002	− 0.88 (− 1.59 to − 0.16); − 0.08; 0.016
	High school	− 0.57 (− 0.85 to − 0.29); − 0.14; < 0.001	− 0.74 (− 1.19 to − 0.29); − 0.12; < 0.001	1.12 (0.76 to 1.48); 0.24; < 0.001	− 0.55 (− 0.81 to − 0.29); − 0.15; < 0.001	− 0.85 (− 1.56 to − 0.14); − 0.08; 0.020
	College	− 0.56 (− 0.88 to − 0.25); − 0.11; < 0.001	− 0.85 (− 1.36 to − 0.34); − 0.10; < 0.001	1.54 (1.1 to 1.98); 0.26; < 0.001	− 0.75 (− 1.04 to − 0.46); − 0.16; < 0.001	− 0.68 (− 1.48 to 0.12); − 0.05; 0.096
R-squared		0.159	0.108	0.127	0.060	0.138

*B* regression coefficient, *CI* confidence interval, *Bs* standardized regression coefficient, *BMI* body mass index**,** *NR* not retained in final model.

*The design effect of a cluster sampling approach was taken into consideration and adjusted for in the calculation of 95% CIs (linearized standard error).

## Discussion

The present study is the first population-based study to determine the association VI and mental disorders using a large sample size. As mentioned earlier, various studies reported a significant association between VI and mental disorders^26^ including depression^11^, anxiety^15^, risk of suicide^16^, and cognitive impairment^17^. Mental disorders are a major public health concern in the elderly population, which can lead to suffering, family disruption, disability, worsening of many medical illnesses, and increased mortality^27^. This is while VI also increases with advancing age in the general population^1^ and its coincidence with mental disorders can impose a greater burden on society by causing functional loss, a sense of loneliness, and mortality^28^. It should be noted that most mental disorders and many ocular diseases responsible for visual loss are treatable in the case of early identification. Therefore, early identification of people at risk with timely intervention can be effective in the well-being of the elderly^29^.

VI was significantly associated with depression in the present study and this is in line with several previous reports^26,28–34^. This issue is important to some extent which estimates indicate that one-third of people with VI experience depression or anxiety^35^. The statistical model of the present study showed that the association between VI and depression is significant even after controlling for confounding and background variables. Abdolalizadeh and Falavarjani observed a bidirectional association between VI and depression^36^. Frank et al. reported that adults with self-reported VI had higher odds of developing depression in the future, while those who had baseline depression were more likely to report VI in the future^37^. Furthermore, people with VI are more likely to experience functional problems that reduce personal independence and subsequently lead to mental disorders including depression^31^.

Some studies reported that the more severe the VI, the more severe the depression^34,38^; but other studies have shown that the severity of VI is of less importance and VI can be related to depression regardless of its severity^8,11^. Although many studies in this field were cross-sectional and could not determine the temporal association between VI and depression due to the bidirectional association between these two factors, VI has been suggested to be an independent risk factor for depression^34^. In a review article by Virgili et al., the adjusted odds ratio of developing depression in people with VI was 1.75 in surveys, 1.17 in examinations, and 2.47 in statistical databases^39^; this finding can be explained based on the fact that a significant percentage of patients with depression do not participate in examinations and surveys indicating the need for population-based studies and special planning for this group.

In the present study, the depression coefficient in people with VI was reduced by controlling background and confounding factors; a similar finding was found in the study by Choi et al. In the Choi et al. study, crude analysis showed an odds ratio of 1.22 for the association between VI and depression which decreased to 1.19 after adjusting for age, sex, income, region of residence, hypertension, diabetes, and dyslipidemia^30^. This finding indicates the necessity of a comprehensive look at a set of factors for the correct interpretation of depression in a person with VI; age is one of the main influential factors. A study by Brunes and Heir showed that the rate of major depression decreases with age after 36 years^40^. Other important factors in this regard are the better acceptance of VI and the use of coping strategies^41^.

The results of the present study showed increased anxiety in the presence of VI. Limited studies examined the association between VI and anxiety^31,33,35,42,43^ indicating visually impaired individuals experience a higher level of anxiety. In a study by Jampel et al. 35% of patients recently diagnosed with glaucoma reported some degree of nervousness, anxiety, or stress; the noteworthy point is that none of these patients had severe vision loss, and anxiety was not related to the severity of VI^44^. In explaining this finding, it should be said that worry about living with a disability or being forced to use coping strategies can cause anxiety, even when severe VI has not yet developed^18^. One of the findings of the present study is the lower level of anxiety in blind people compared to those with VI; this finding was also observed in the study by Riazi et al.^45^, and could be explained by lower participation in society and social activities; so, the anxiety related to social issues would be lower in blind people^45^.

In the present study, the coefficient of anxiety in patients with VI was 0.513 while controlling for background and confounding factors. Evans et al. reported that by controlling for age and sex, VI increases the odds of anxiety by 1.4 times^31^. In fact, anxiety could be proposed as a psychological consequence of vision loss^35^, which significantly affects the daily activities of a person with VI^18^. It have been shown that psychological reactions to stressful conditions are age-dependent^18^, Besides, studies indicate that the level of anxiety for people who experience vision loss at a young age is significantly higher than that of older counterparts^46^. A study on Japanese patients with visual impairment similarly reported that the level of anxiety decreases with advancing age^42^.

Some studies have shown that the level of anxiety in the elderly depends on the type of pathology. In a study by Eramudugolla et al., anxiety symptoms were developed in patients with senile cataracts, while this finding was not observed in other pathologies such as glaucoma and age-related macular degeneration (AMD)^47^. It seems that individuals with moderate VI constantly fear further deterioration of vision up to blindness; In contrast, those with severe VI have reached a better acceptance of the condition^35,42^. Psychological costs have also been shown to be higher in patients with milder levels of VI^42^. There are some conflicting reports regarding anxiety in patients with VI. In contrast to the study by Eramudugolla et al.^47^, some other studies found a high level of anxiety in patients with AMD^48^ and primary open-angle glaucoma^15^. Factors such as different symptom evaluation methods and different disease severities may explain this discrepancy.

VI was associated with decreased physical activities in the present study. This issue has been addressed in various studies. It has been shown that people with VI face significant problems in performing basic activities of daily living such as eating, wearing clothes, writing, and even simple daily communication^49^. In addition to physical strength, physical activities require proper visual function in terms of visual acuity, contrast sensitivity, and visual field, which are affected by various ocular pathologies^50^. Therefore, different levels of physical function limitations are expected in patients with VI depending on the severity of the underlying ocular pathology^51^. It should be noted that the regression coefficient of the physical subscale in the crude model was higher than the adjusted models; the confounding effect of age is probably the main reason considering that aging itself is associated with a decreased physical activity due to a decrease in physical strength^52^. In our study, social activities did not have a statistically significant association with VI in the final linear regression model. Although this association has been reported in previous studies^53,54^. In the Blue Mountains Eye Study, VI negatively affected social functioning and individual independence in the elderly^54^. As explained earlier, VI leads to depression and anxiety and also reduces the physical activities of the affected person. Elmer and Stadtfeld stated that depressive symptoms lead to social isolation^55^. On the other hand, the social perception of a person with a disability is one of the main factors affecting non-participation in physical activities^56^. Although the elderly can respond appropriately to environmental challenges, when the demand for daily tasks increases and the person’s vision is impaired, physical limitations can affect the quality of life^32^. This is while the simultaneity of aging and VI further reduces a person’s ability level compared to when there is only one of these problems (aging or VI); which in turn can lead to unhappiness, hopelessness, and worthlessness^32,57^. The discrepancy between the present study and previous studies may be due to cultural and social differences, which require further studies^57^.

The present study has strengths and limitations. The tool used to measure the study outcomes was different from many other studies; therefore, the findings should be compared with caution; Besides, although the questionnaire information was recorded by an expert psychologist, nevertheless, the evaluation of the mental state of the people was done only based on the score of the questionnaire. Due to the cross-sectional design, it was not possible to determine the temporal precedence between mental disorders and VI. We only controlled background and predisposing variables and did not consider the role of factors affecting VI such as refractive error, cataracts, AMD, and other diseases. Although controlling for other ocular diseases could have provided a more accurate estimate of the association between mental disorders and VI, it was not among the objectives of this study, it is recommended that these issues be investigated in future studies. The large sample size, sampling and careful examination by an expert and trained team, as well as the control of many effective variables, are the strengths of the present study, which provides the possibility of generalizing the obtained results to the society of elderly population in Iran.

## Conclusion

In general, VI is associated with an increase in physical symptoms, anxiety, and depression. However, the majority of these psychological disorders can be attributed to factors other than VI, especially functional impairment. Although it was not possible to determine the temporal sequence between VI and mental disorders in this cross-sectional study, one possible scenario is that individuals with VI are more likely to experience functional problems, which in turn lead to mental disorders. This finding emphasizes the importance of a broader view of the aging process and the elderly.

## Data Availability

The datasets used and/or analyzed during the current study available from the corresponding author on reasonable request.
